# A Rare Case of Recurrent Delayed Massive Hematuria Due to Arterial Bleeding Following Water Vapor Energy Therapy for Benign Prostatic Hyperplasia

**DOI:** 10.7759/cureus.102124

**Published:** 2026-01-23

**Authors:** Seiya Shiramizu, Sato Hiroaki, Yuto Tsubonuma, Rieko Kimuro, Katsuyoshi Higashijima, Kazunobu Aramaki, Naohiro Fujimoto

**Affiliations:** 1 Urology, Local Incorporated Administrative Agency Kurate Hospital, Kurate, JPN; 2 Urology, University of Occupational and Environmental Health, Kitakyushu, JPN

**Keywords:** arterial bleeding, bph, delayed bleeding, rezūm, wave

## Abstract

Water vapor energy therapy (WAVE, Rezūm™) is a minimally invasive surgical therapy for benign prostatic hyperplasia (BPH) that is associated with favorable efficacy and safety. Clinically significant bleeding requiring transfusion or endoscopic intervention is rare.

A 77-year-old man with BPH developed recurrent delayed massive hematuria following WAVE. Although the initial postoperative course was uneventful, he presented on postoperative day 7 with bladder tamponade, managed with clot evacuation. On postoperative day 21, after catheter removal, he experienced acute urinary retention and hypotension, accompanied by gross hematuria. Endoscopic evacuation and coagulation of arterial bleeding from the median lobe achieved hemostasis, and the patient recovered without further recurrence.

To the best of our knowledge, based on a review of the currently available English-language literature, no prior cases of delayed arterial hemorrhage requiring intervention after WAVE have been reported. Clinicians should remain vigilant for this possibility, and patients should be informed of this risk.

## Introduction

Benign prostatic hyperplasia (BPH) is one of the most common urological diseases in aging men. Because many patients are elderly and have multiple comorbidities, minimally invasive surgical therapies are preferred. Water vapor energy therapy (WAVE, Rezūm™) has emerged as an effective and safe option, providing durable improvements in lower urinary tract symptoms and urinary flow rates [1]. Hematuria is frequently observed after WAVE but is generally mild and self-limiting. Cases requiring endoscopic coagulation or transfusion are extremely rare [2,3]. To date, there have been no reports of delayed arterial hemorrhage following WAVE. We report here the first case of recurrent delayed massive hematuria due to arterial bleeding after this procedure.

## Case presentation

A 77-year-old Japanese man with a history of polycythemia vera treated with aspirin (100 mg daily) and type 2 diabetes managed with linagliptin (5 mg daily) presented with urinary retention due to BPH. He had been on naftopidil (50 mg daily) for seven years. A urethral catheter was inserted, and three weeks later, WAVE was performed while continuing aspirin. Preoperative evaluation revealed a prostate volume of 32 mL and an intravesical prostatic protrusion of 24 mm. WAVE was performed under general anesthesia, with two injections administered on each side of the prostatic urethra and an additional injection delivered into the hypertrophic median lobe at the seven o’clock position. The initial postoperative course was uneventful, with no gross hematuria. He was discharged on postoperative day 4 with the catheter in place. On postoperative day 7, he presented with bladder tamponade. Blood clots were evacuated through bladder irrigation, after which hematuria subsided. On postoperative day 21, the catheter was removed; residual urine volume was 57 mL, and voiding was satisfactory. However, eight hours later, he developed acute urinary retention and was readmitted.

**Figure 1:**
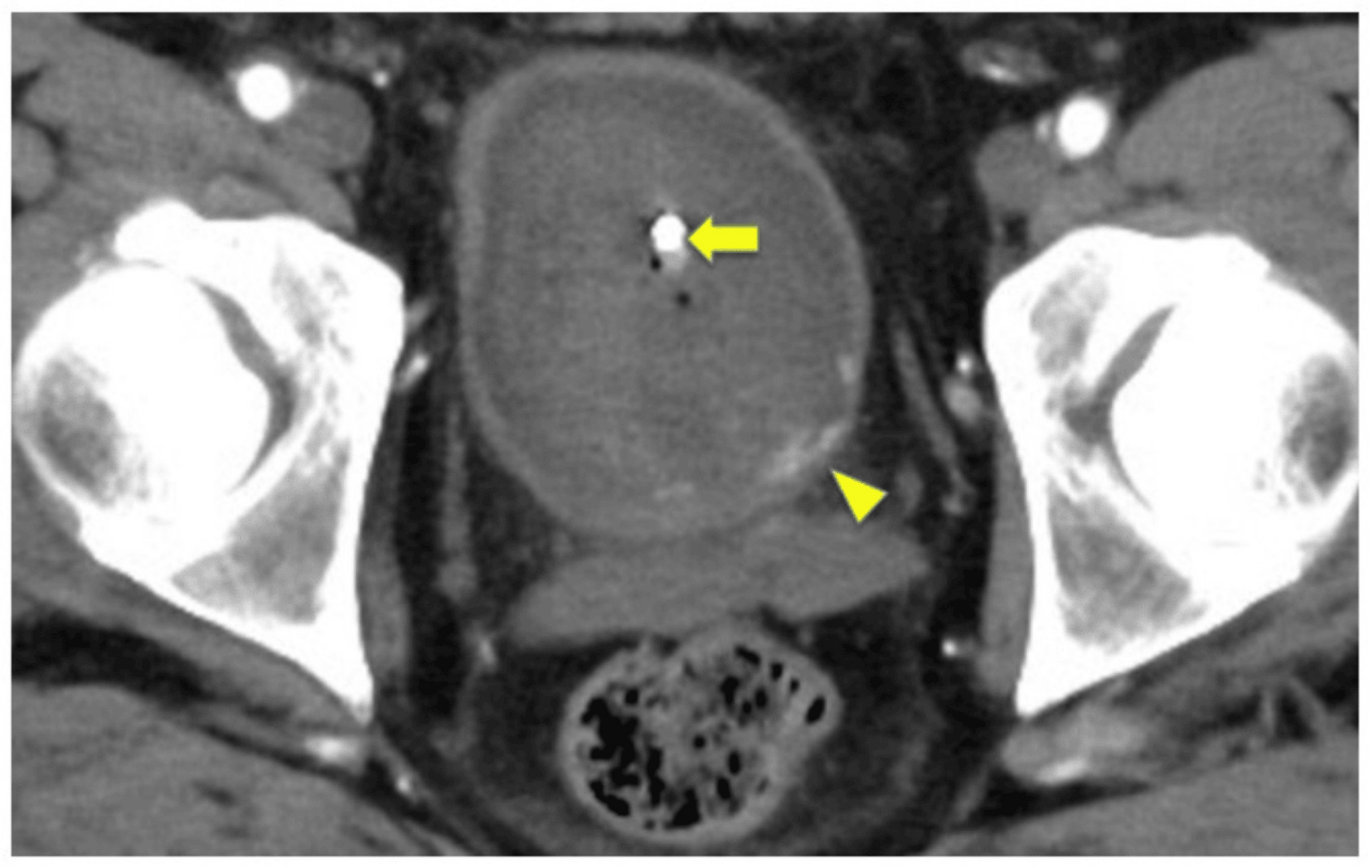
Contrast-enhanced pelvic CT (arterial phase). Contrast extravasation (arrowhead) is seen from the bladder neck at the five o’clock position; the catheter is indicated by the arrow.

**Figure 2:**
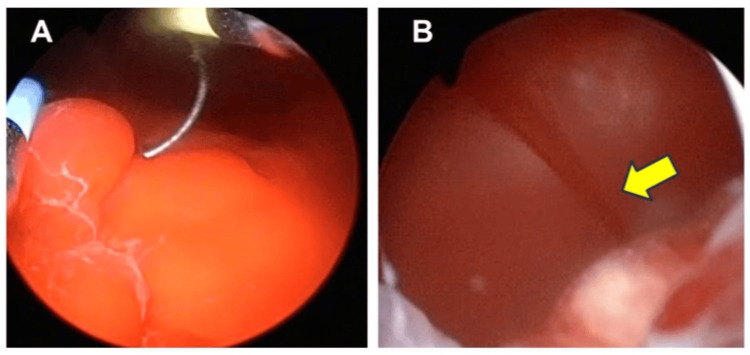
Cystoscopic findings. Bright red clot on the bladder neck (A) and active arterial bleeding from the median lobe at the five o’clock (B).

**Figure 3:**
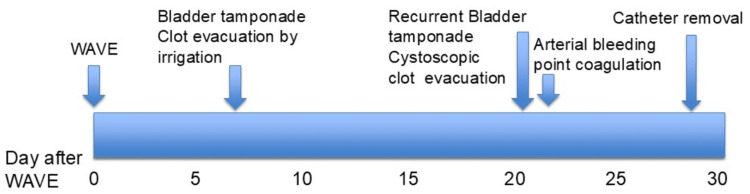
Timeline of clinical course after water vapor energy therapy (WAVE).

On admission, blood pressure was 91/60 mmHg (his baseline was 132/77 mmHg), and pulse rate was 102 beats per minute (bpm) (his baseline was 78 bpm). The patient developed rapidly progressive anemia (Table 1). A Foley catheter was inserted, draining bright red urine suspicious for arterial bleeding. Emergent cystoscopy revealed a large volume of clots, but no active bleeding was initially detected. Aspirin was discontinued. The following day, recurrent gross hematuria occurred. Contrast-enhanced CT in the arterial phase demonstrated contrast extravasation from the bladder neck at the five o’clock position (Figure 1). Hemoglobin dropped to 7.4 g/dL and red blood cell (RBC) count to 3.41×10⁶/μL. He received four units of packed red blood cells and 240 mL of fresh frozen plasma. Repeat cystoscopy confirmed a bright red clot, suspected to be of arterial origin, extending from the bladder neck into the bladder cavity (Figure 2A). Upon the removal of the clot, active arterial spurting hemorrhage was observed in the five o’clock position of the prostatic median lobe toward the bladder lumen (Figure 2B). Electrocautery was applied, achieving complete hemostasis. Thereafter, hematuria resolved. Aspirin was restarted on postoperative day 4 after coagulation. The catheter was removed on day 7, and the patient was discharged on day 8. No further hematuria was observed during follow-up (Figure 3).

**Table 1:** Laboratory findings on admission. Descriptive clinical variables are reported as observed and do not require literature references. PT-INR, prothrombin time-international normalized ratio; APTT, activated partial thromboplastin time

Parameters	On admission	His baseline	Reference
White blood cell count (/μL)	32,180	19,810	3,300-8,600
Red blood cell count (×10^4^/μL)	477	614	435-555
Hemoglobin (g/dL)	10.5	13.4	13.7-16.8
Hematocrit (%)	33.8	42.4	40.7-50.1
Platelet count (×10^3^/μL)	307	232	158-348
PT-INR	1.21	1.14	0.85-1.15
APTT (seconds)	31.7	34.4	23.5-35.5

## Discussion

Delayed hematuria after prostate procedures such as the transurethral resection of the prostate, laser prostatectomy, and photoselective vaporization has been described [4-6]. However, to our knowledge, delayed arterial bleeding after WAVE has not previously been reported.

In our case, immediate postoperative bleeding was minimal, yet recurrent massive hematuria occurred one and three weeks later. Notably, the bleeding site was distinct from the needle puncture sites, suggesting that direct procedural trauma was unlikely. Although no direct pathological confirmation was available, delayed thermal injury to periprostatic vessels induced by convective water vapor energy may represent a plausible mechanism for the observed delayed arterial bleeding. WAVE induces tissue ablation through convective heating, creating coagulative necrosis and subsequent resorption [1]. Experimental studies have demonstrated that vascular smooth muscle contractility and endothelial function are impaired at temperatures as low as 55°C-60°C, leading to reduced vasoreactivity [7]. During WAVE, adjacent tissues can transiently reach 70°C or higher, which may compromise the structural integrity of nearby arteries, predisposing them to delayed rupture [8].

In addition, the presence of polycythemia vera and the continuation of aspirin therapy may have increased the patient’s susceptibility to delayed bleeding and should be considered potential contributing factors in this case.

The management of arterial bleeding after WAVE should follow the general principles used for other iatrogenic prostatic hemorrhages. Endoscopic coagulation is usually effective as first-line therapy. When hemostasis cannot be achieved, prostatic artery embolization represents a minimally invasive alternative with high efficacy in controlling intractable prostatic bleeding [9]. Our case highlights that although hematuria following WAVE is usually self-limiting and mild, clinicians must recognize that rare but severe delayed arterial hemorrhage can occur. Prompt recognition using contrast-enhanced CT and cystoscopy, followed by early multidisciplinary intervention, is essential for optimal patient outcomes.

## Conclusions

We describe a rare case of recurrent delayed massive hematuria following WAVE for BPH. Although extremely rare, delayed arterial bleeding may occur after WAVE, and clinicians should remain aware of this potential complication when counseling patients.
